# Recurrent Labial Herpes Simplex in Pediatric Dentistry: Low-level Laser Therapy as a Treatment Option

**DOI:** 10.5005/jp-journals-10005-1252

**Published:** 2014-08-29

**Authors:** Priscila Stona, Elizabete da Silva Viana, Leandro dos Santos Pires, João Batista Blessmann Weber, Paulo Floriani Kramer

**Affiliations:** Managing Editor, Department of Pediatric Dentistry, Universidade Luterana do Brasil, RS, Brazil; Managing Editor, Department of Pediatric Dentistry, Universidade Luterana do Brasil, RS, Brazil; Managing Editor, Department of Pediatric Dentistry, Universidade Luterana do Brasil, RS, Brazil; Managing Editor, Department of Pediatric Dentistry, Pontificia Universidade Católica do Rio Grande do Sul, RS, Brazil; Managing Editor, Department of Pediatric Dentistry, Universidade Luterana do Brasil, RS, Brazil

**Keywords:** Herpes simplex, Laser therapy, Pediatric dentistry

## Abstract

Recurrent labial herpes simplex is a pathology of viral origin that is frequently observed in children. The signs and symptoms are uncomfortable and, in many cases, the efficacy of treatment is unproven. However, several studies have demonstrated good results from the use of low-level laser therapy (LLLT), primarily due to acceleration of the healing process and pain relief, which make it a promising resource for use with this pathology. This paper describes a clinical case of a 7-year-old patient affected by this pathology and the therapeutic resolution proposed.

**How to cite this article:** Stona P, da Silva Viana E, dos Santos Pires L, Weber JBB, Kramer PF. Recurrent Labial Herpes Simplex in Pediatric Dentistry: Low-level Laser Therapy as a Treatment Option. Int J Clin Pediatr Dent 2014;7(2):140-143.

## INTRODUCTION

Herpes is a viral infection caused by the herpes simplex virus (HSV-1). It generally presents as a primary lesion, with periods of latency and a tendency to relapse.^1^ Manifestations can vary from a mild period of fever to a complete loss of appetite as a response to the ulcers that appear after vesicles or blisters burst.

The incidence of primary infections increases after 6 months of age, when the HSV antibodies acquired from the mother disappear, and reaches a peak between 2 and 3 years. Nevertheless, new cases can appear in older children, adolescents and previously uninfected adults.^2^

After the resolution of the lesions, the virus moves through the nerve endings and establishes a latent state at sensory ganglia, most commonly the trigeminal ganglion.^3^ During latency, the virus has no impact on infected cells and can be reactivated several times during the host’s life.^4^ The virus’s reactivation may be triggered by internal or external stimuli, such as stress, immunosuppression, fever, menstruation and exposure to sunlight, among others.^3-5^

Clinically, the viral infection begins with prodromal signs or symptoms, such as mild itching that progresses rapidly to blisters or vesicles. After these burst, erosions and ulcers appear and, finally, scabs appear at the end of the herpes cycle.^6,7^ During the first days, pain can be sufficient to cause a lack of appetite, provoking intense systemic involvement. The virus is disseminated when children are irritated by the itching and scratch, bursting the blisters and spreading the virus, very often into the eyes and nose, and other parts of the body.

Treatment for herpes is usually symptomatic and consists of a soft diet, ample fluid intake, analgesics and antipyretics aimed at relieving discomfort.^8^ Antiviral agents (acyclovir) are indicated for immunosuppressed patients, in cases of severe ulcers or when recurrence is frequent. When the suspension is administered before the onset of the vesicles or during the first days of symptomology, clinical resolution is significantly accelerated.^7,9^

However, scientific evidence has highlighted the ineffectiveness of topical medications and other complementary methods. Moreover, the intermittent administration of acyclovir can promote drug resistance.^7^ Low-level lasers have nevertheless demonstrated great potential for clinical improvement of painful herpes sores by accelerating the process of healing by biostimulation of tissues and production of pain relief.^8^ Lasers are capable of curing herpes sores more quickly, reducing healing time, and they can also reduce the recurrence of sores.^10^

The objective of this article is to demonstrate the use of a low-level laser therapy (LLLT) as a treatment option for recurrent labial herpes sores in pediatric dentistry.

## CASE REPORTS AND RESULTS

A 7-year-old female presented for dental treatment complaining of difficulty in chewing, intense pain on left side of lower lip, lack of appetite, indisposition and difficulty sleeping at night.

**Fig. 1:**
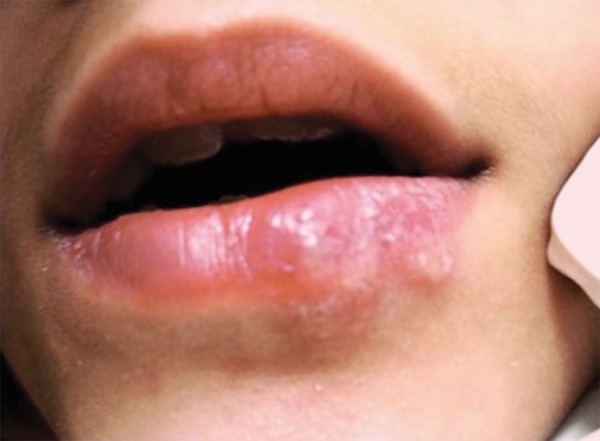
Initial appearance of sore

During physical examination, mild submandibular glandular swelling was observed on the left-hand side, with mildly painful symptomology. There was also a large area with blisters, extending from the vermilion border to the lower part of the lip (Fig. 1). The patient reported that the sores had appeared within the previous 24 hours. When asked, the patient reported an itching sensation. According to her mother, this was not the first episode. A diagnosis of recurrent labial herpes simplex was made.

**Fig. 2:**
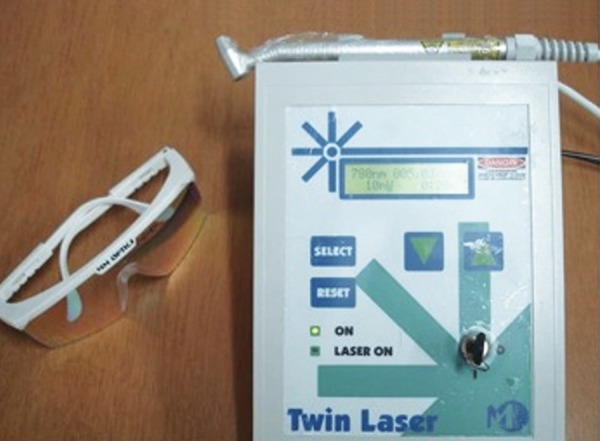
Low-intensity laser equipment utilized

**Fig. 3:**
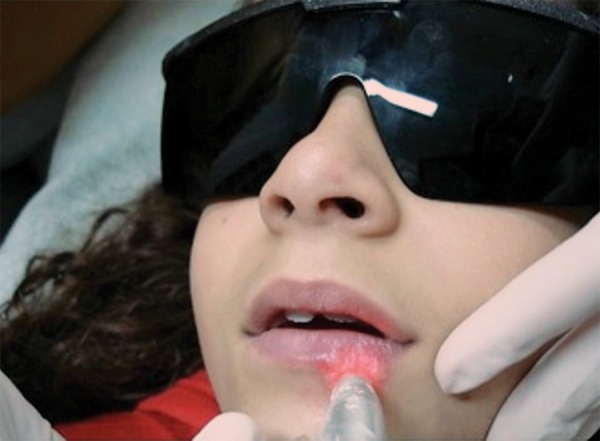
Application of laser light on herpes sore. Use of safety equipment

The treatment proposed and implemented was a series of three irradiations of a low-level laser (GaAlAs) in continuous emission mode (5 J/cm^2^) on the herpes sores, performed every 24 hours. An infrared diode laser (Twin Laser^®^, MM Optics, São Carlos, Brazil) was employed at a wavelength of 780 nm (70 mW) for 1 minute and 20 seconds (80 seconds) at four points on the herpes sores (total dose per session = 20 J/cm^2^). During the applications, protective barriers were employed, and safety glasses of the color and optical density recommended by the manufacturer were used (Figs 2 and 3).

**Fig. 4:**
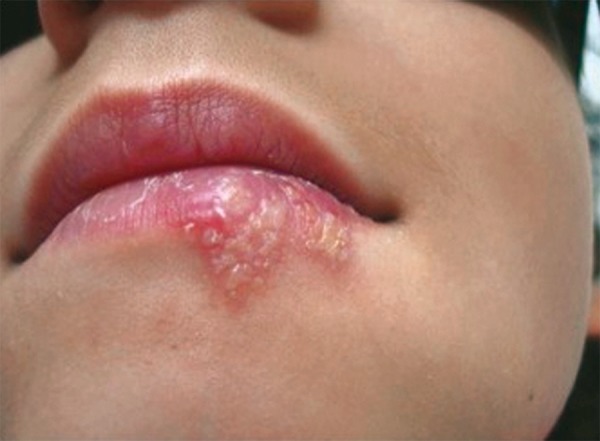
Second day of application. Observe that hyperemia is reduced around the sore and the liquid content of the vesicles has also decreased

At the second session of irradiation, the patient no longer exhibited painful symptomology or itching, nor any functional limitations. The patient did not report prostration or lack of appetite. It could be observed that the area of greatest redness had disappeared and that the liquid content of the blisters had decreased. Other areas had already moved into the scab phase, indicating the start of healing. There was a reduction in the clinical extent of the lesion (Fig. 4).

**Fig. 5:**
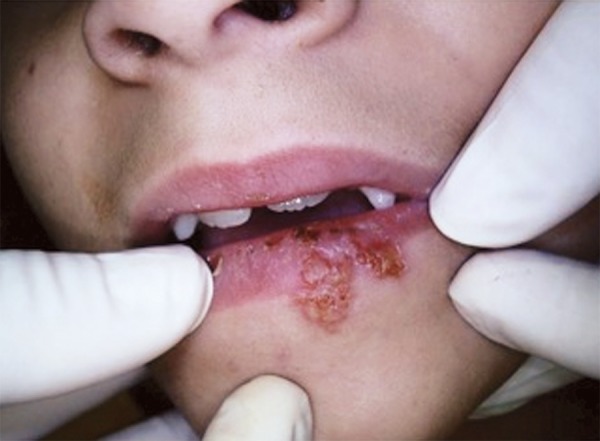
Third day of low-intensity laser application. Observe that the healing process has been accelerated and the blisters have reached the scab stage

**Fig. 6:**
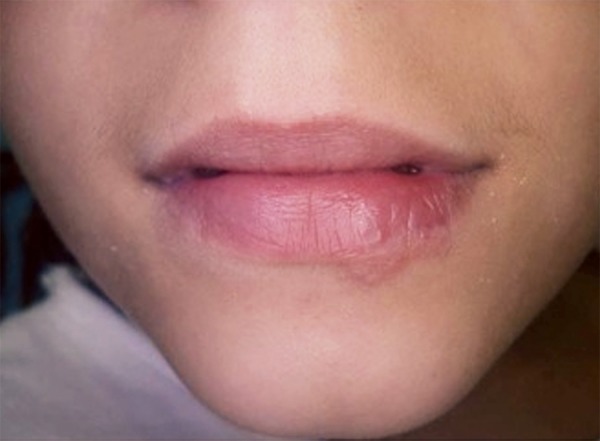
Ten days after the first application. No more active herpes areas are observed, and the mucosa of the lower lip has nearly recovered

At the third and last irradiation, almost all of the blisters were in the scab phase, and the case remained asymptomatic (Fig. 5). Ten days after the first application, the patient returned for follow-up, and no fresh activation of the virus or herpetic whitlow was observed (Fig. 6). Furthermore, the patient’s mucosa and epithelium were healing well with no symptomology.

## DISCUSSION

Many attempts have been made to develop treatment or preventive methods to minimize the severity of herpes simplex virus (HSV). Treatments that have been proposed to date have not met with significant success, whether because of difficulties in controlling the application of topical medications or because of the need to initiate treatment at the point when the patient does not yet exhibit visible clinical signs.^3,11^ In the case report described, the patient had already undergone other treatments without perceiving any reduction in the duration of manifestations or in their recurrence. Laser light is a form of radiation that does not ionize, is highly concentrated and, which, on contact with different types of tissue and depending on the type of laser, results in photochemical, photoelectric, photothermal, or photomechanical effects. Laser radiation is not invasive, is well tolerated by tissues, does not have mutagenic effects, and can be used repeatedly without risk.^12^

Low-level laser therapy has many physiological effects, such as anti-inflammatory and analgesic properties and stimulation of wound healing.^3,8^ Low-level lasers or healing lasers, as they are also known as, use an energy level below 500 W and do not cause temperature elevation in the target tissue.^12^ These lasers also produce a photobiological or photochemical effect on the target tissue.^12,13^

Lacour,^14^ in a double-blind placebo-controlled study, detected a mean increase in HSV latency of 4 to 37.5 weeks in a group treated with laser light, while a placebo group demonstrated a mean increase of 3 weeks. Kotlow^12^ reported that most of the herpetic lesions of a 10-year-old boy were resolved after the use of LLLT at a follow-up visit 4 days after the application with no discomfort at the time. Navarro et al^8^ and Bello-Silva et al^10^ concluded in different studies that laser therapy should be considered as an alternative treatment in HSV-1 infection. Eduardo et al^3^ in a 3-year follow-up study using LLLT for preventive treatment of recurrent labial herpes found that not only were outbreaks less frequent and the healing process faster but also the intensity of the infection’s symptoms was milder than ever.

In pediatric dental practice, the use of lasers has offered increased comfort as a result of its biomodulation effects and rapidity in initiating the healing process of the cycle and remission of symptoms in the treatment of recurrent labial herpes. Nevertheless, the mechanism by which the monochromatic light acts on tissues, cells, organelles, and cellular substances is not yet fully elucidated and requires wider-ranging studies.

## CONCLUSION

Low-level laser therapy is an important alternative for treatment of recurrent labial herpes simplex in pediatric dentistry. It can relieve painful symptoms and accelerate the healing process.
